# Design, synthesis and evaluation of ^18^F-labeled cationic carbonic anhydrase IX inhibitors for PET imaging

**DOI:** 10.1080/14756366.2017.1308928

**Published:** 2017-04-07

**Authors:** Zhengxing Zhang, Joseph Lau, Chengcheng Zhang, Nadine Colpo, Alessio Nocentini, Claudiu T. Supuran, François Bénard, Kuo-Shyan Lin

**Affiliations:** a Department of Molecular Oncology, BC Cancer Agency, Vancouver, British Columbia, Canada;; b Department of Neurofarba, Section of Pharmaceutical and Nutriceutical Sciences, Università Degli Studi Di Firenze, Florence, Italy;; c Department of Functional Imaging, BC Cancer Agency, Vancouver, British Columbia, Canada;; d Department of Radiology, University of British Columbia, Vancouver, British Columbia, Canada

**Keywords:** Carbonic anhydrase IX, fluorine-18, molecular imaging, positron emission tomography

## Abstract

Carbonic anhydrase IX (CA-IX) is a marker for tumor hypoxia, and its expression is negatively correlated with patient survival. CA-IX represents a potential target for eliminating hypoxic cancers. We synthesized fluorinated cationic sulfonamide inhibitors **1**–**3** designed to target CA-IX. The binding affinity for CA-IX ranged from 0.22 to 0.96 μM. We evaluated compound **2** as a diagnostic PET imaging agent. Compound **2** was radiolabeled with ^18^F in 10 ± 4% decay-corrected radiochemical yield with 85.1 ± 70.3 GBq/μmol specific activity and >98% radiochemical purity. ^18^F-labeled **2** was stable in mouse plasma at 37 °C after 1 h incubation. PET/CT imaging was conducted at 1 h post-injection in a human colorectal cancer xenograft model. ^18^F-labeled **2** cleared through hepatobiliary and renal pathways. Tumor uptake was approximately 0.41 ± 0.06% ID/g, with a tumor-to-muscle ratio of 1.99 ± 0.25. Subsequently, tumor xenografts were visualized with moderate contrast. This study demonstrates the use of a cationic motif for conferring isoform selectively for CA-IX imaging agents.

## Introduction

Tumor cells cycle through stages of being well-oxygenated or oxygen-deprived. Hypoxia occurs when oxygen level of the microenvironment is unable to sustain the metabolic demands of a growing tumor^1^. Regardless of size, stage, grade, or histology, all solid tumors are susceptible to hypoxia^2^. Although hypoxia may initially limit growth potential, it also promotes and regulates biological processes like angiogenesis, invasiveness, metastasis, metabolism, and genomic instability^3^. Moreover, hypoxia confers resistance and insensitivity to conventional chemotherapy and radiotherapy^3^^,^^4^. As such, therapeutic strategies targeting hypoxia and/or components of the hypoxia-induced signaling pathway are consistently being explored^3^. Inherently, there is a need to develop companion diagnostics that can be used for patient stratification or treatment response assessment.

An established surrogate marker for hypoxia is carbonic anhydrase IX (CA-IX). CA-IX is the protein that is most strongly upregulated by hypoxia and hypoxia-inducible factor 1α (HIF-1α)^5^. CA-IX is 1 of 15 unique but closely related zinc metalloenzymes^6^. Of the pertinent isoforms, CA-IX is preferentially expressed in solid malignancies to maintain intracellular pH homeostasis in concert with ion transporter systems^7–11^. In preclinical studies, attenuation of CA-IX activity by small molecule inhibitors has been shown to be efficacious in reducing primary tumor growth and distant metastases^12–15^. CA-IX inhibitors have also been used as delivery vectors of cytotoxic payloads to target tumor cells within hypoxic niches^16–18^. Complementing these therapeutic efforts has been the development of CA-IX radiotracers derived from inhibitors for positron emission tomography (PET) and single photon emission computed tomography (SPECT) applications^19–31^. PET and SPECT can generate images of high resolution and sensitivity, while providing quantitative information on drug target expression.

**Figure 1.**
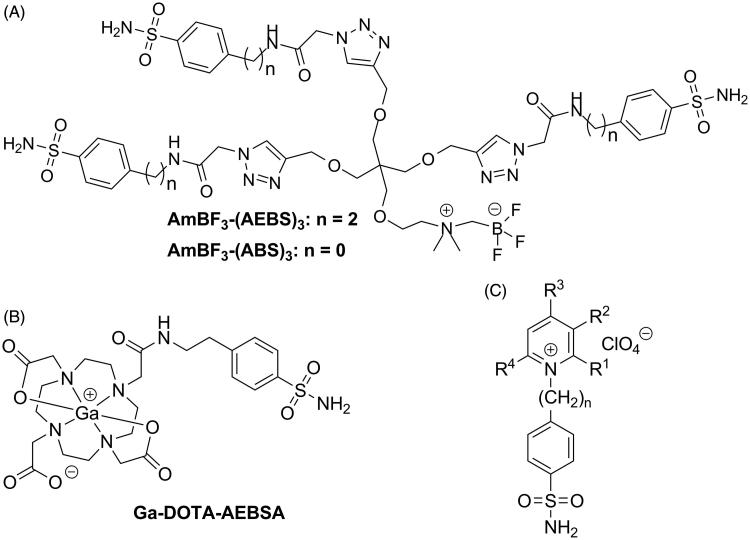
Reported sulfonamide derivatives that are CA-IX selective agents. (A) Trimeric AmBF_3_-(AEBS)_3_ and AmBF_3_-(ABS)_3_, (B) Ga-DOTA-AEBSA, and (C) cationic sulfonamide derivatives.

CA-IX is an attractive target because of its pathological expression in cancers, as well as the fact that it resides as a cell surface transmembrane protein^6^. However, the highly conserved catalytic domain shared by CA isoforms poses as a challenge for synthesizing CA-IX selective imaging agents^6^. Off-target binding to intracellular CAs, notably CA-I and CA-II expressed in erythrocytes, can reduce tumor targeting and contrast ratios^6^. Therefore, strategies to confer CA-IX selectivity for small molecule inhibitors have focused on limiting transport across the plasma membrane. Previously, our research group reported two approaches for sulfonamide-based CA-IX imaging agents that successfully targeted human colorectal cancer xenografts. In one approach, we leveraged a multimeric design to synthesize ^18^F-labeled trivalent tracers (Figure 1(A)) that were of sufficient bulk (MW >1 kDa) to be cell-impermeable^26^. In the other approach, we conjugated pharmacophores to different polyaminocarboxylate chelators for ^68^Ga-radiolabeling (Figure 1(B))^27^. The hydrophilicity of the metal/chelator complex facilitated selective targeting of CA-IX *in vivo*.

**Figure 2.**
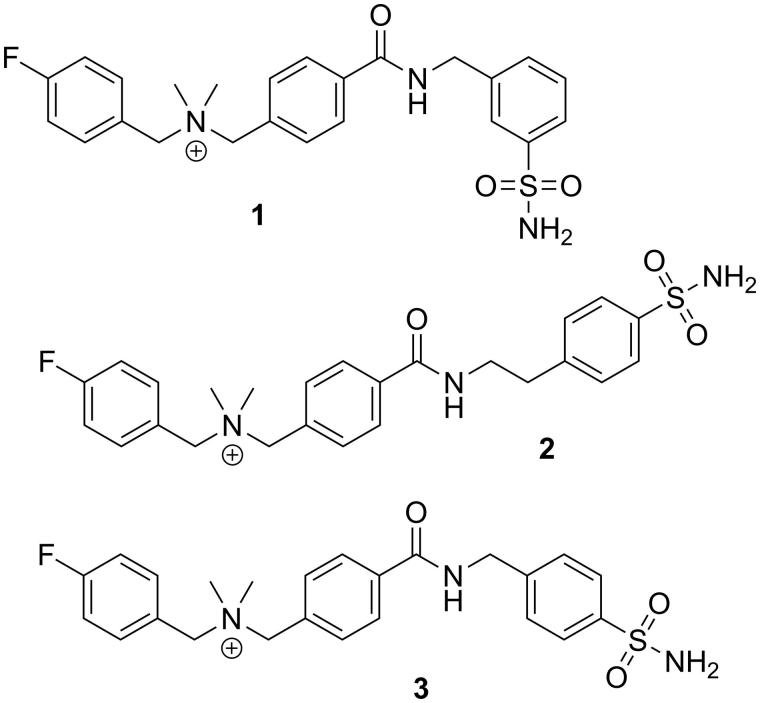
Chemical structures of three cationic carbonic anhydrase IX inhibitors evaluated in this study.

In addition to factors like size and hydrophilicity, the overall charge of a molecule can modulate the property to permeate through the plasma membrane. Previous work published by Casey et al.^32^ demonstrated that cationic sulfonamide derivatives showed favorable CA-IX inhibition profiles (Figure 1(C)). As charged molecules, these inhibitors have limited transcellular uptake^33^. In this paper, we investigated if fluorinated sulfonamide derivatives engrafted with a cationic motif can be used as potential PET tracers for imaging CA-IX expression in tumors (Figure 2).

## Materials and methods

### Chemicals and instrumentation

All chemicals and solvents were obtained from commercial sources, and used without further purification. Triethylammonium phosphate buffer (TEA-PB, pH 7.29) was prepared by titrating a solution of triethylamine (8 mL) in deionized water (1 L) with *o*-phosphoric acid monitored using a Denver Instrument (Bohemia, NY) UltraBasic Benchtop pH meter. Proton NMR spectra were obtained using a Bruker (Billerica, MA) Avance 400inv Spectrometer, and are reported in parts per million. Mass analyses were performed using an AB SCIEX (Framingham, MA) 4000 QTRAP mass spectrometer system with an ESI ion source. Melting points were taken on a Fisher–Johns melting point apparatus (Fisher Scientific, Pittsburgh, PA) and were uncorrected. The quaternary methyl ammonium (QMA) anion exchange and C18 light Sep-Pak cartridges (1 cm^3^, 50 mg) were obtained from Waters (Milford, MA). Purification and quality control of ^18^F-labeled **2** were performed on an Agilent (Santa Clara, CA) HPLC system equipped with a model 1200 quaternary pump, a model 1200 UV absorbance detector, and a Bioscan (Washington, DC) NaI scintillation detector. The radio-detector was connected to a Bioscan B-FC-1000 Flow-count system, and the output from the Bioscan Flow-count system was fed into an Agilent 35900E Interface, which converted the analog signal to digital signal. The operation of the Agilent HPLC system was controlled using the Agilent ChemStation software. The HPLC columns used were a semi-preparative column (Phenomenex C18, 5 μ, 250 × 10 mm) and an analytical column (Phenomenex C18, 5 μ, 250 × 4.6 mm). ^18^F-Fluoride was produced by the ^18^O(p, n)^18^F reaction using an Advanced Cyclotron Systems Inc. (Richmond, BC, Canada) TR19 cyclotron. Radioactivity of ^18^F-labeled **2** was measured using a Capintec (Ramsey, NJ) CRC^®^-25 R/W dose calibrator, and the radioactivity of mouse tissues collected from biodistribution studies were counted using using a Perkin Elmer (Waltham, MA) Wizard2 2480 automatic gamma counter. PET imaging experiments were conducted using a Siemens (Knoxville, TN) Inveon microPET/CT scanner.

### Syntheses of precursors and standards

#### 2,3,5,6-Tetrafluorophenyl 4-[(dimethylamino) methyl] benzoate (4)

A mixture of 4-[(dimethylamino) methyl] benzoic acid hydrochloride (2.37 g, 11 mmol), 2,3,5,6-tetrafluorophenol (1.99 g, 12 mmol), and *N*,*N*-diisopropylethylamine (1.42 g, 11 mmol) in dichloromethane (80 mL) was added *N*,*N*′-dicyclohexylcarbodiimide (2.06 g, 10 mmol). After stirring at room temperature for 27 h, the reaction mixture was filtered, and the filtrate was extracted with 2 M NaOH aqueous solution (100 mL). The organic phase was dried with anhydrous magnesium sulfate, and evaporated under reduced pressure. The residue was dissolved in 1:1 diethyl ether/hexane (100 mL), and the insoluble crystals were removed by filtration. The filtrate was concentrated under reduced pressure to yield **4** as colorless oil (3.25 g, 99%). ^1 ^H NMR (300 MHz, CDCl_3_) δ 8.17 (d, *J* = 8.3 Hz, 2H), 7.50 (d, *J* = 8.3 Hz, 2H), 7.04 (tt, *J* = 9.9, 7.0 Hz, 1H), 3.53 (s, 2H), 2.27 (s, 6H). MS (ESI) calculated for C_16_H_13_F_4_NO_2_ 327.1, found (M + H)^+^ 328.0.

#### *N*,*N*-dimethyl-*N*-(4-fluoro)benzyl-4-[(2,3,5,6-tetrafluorophenoxy) carbonyl] benzylammonium bromide (5)

4-Fluorobenzyl bromide (236 μL, 359 mg, 1.9 mmol) was added to a solution of **4** in acetonitrile (6 mL). The reaction mixture was stirred at room temperature for 3 days. The precipitated product was filtered, and washed with diethyl ether (2 mL ×3) to yield **5** as white powder (852 mg, 87%). ^1 ^H NMR (300 MHz, DMSO) δ 8.33 (d, *J* = 8.3 Hz, 2H), 8.05 (tt, *J* = 10.9, 7.5 Hz, 1H), 7.89 (d, *J* = 8.3 Hz, 2H), 7.67 (dd, *J* = 8.6, 5.5 Hz, 2H), 7.38 (t, *J* = 8.8 Hz, 2H), 4.74 (s, 2H), 4.65 (s, 2H), 2.93 (s, 6H). MS (ESI) calculated for C_23_H_19_F_5_NO_2_^+^ 436.1, found (M)^+^ 435.8. Melting point: 198 °C.

#### *N*-4-[[[3-(aminosulfonyl) benzyl] amino] carbonyl] benzyl-*N*,*N*-dimethyl-4-fluorobenzylammonium bromide (1)

A solution of **5** (155 mg, 0.3 mmol) and 3-(aminomethyl) benzenesulfonamide (80 mg, 0.43 mmol) in methanol (5 mL) was stirred at room temperature for 2 days. The solvent was removed by heating at 70 °C. Tetrahydrofuran (2 mL) was added to the residue, and the resulting mixture was heated at 70 °C for 2 min. The insoluble product was isolated by filtration to yield **1** as white solid (156 mg, 97%). ^1^H NMR (300 MHz, DMSO) δ 9.32 (t, *J* = 5.9 Hz, 1H), 8.04 (d, *J* = 8.2 Hz, 2H), 7.78 (s, 1H), 7.75–7.68 (m, 3H), 7.68–7.63 (m, 2H), 7.58–7.51 (m, 2H), 7.44–7.29 (m, 4H), 4.65 (s, 2H), 4.64 (s, 2H), 4.57 (d, *J* = 5.8 Hz, 2H), 2.89 (s, 6H). MS (ESI) calculated for C_24_H_27_FN_3_O_3_S^+^ 456.2, found (M)^+^ 456.2. Melting point: 226 °C.

#### *N*-4-[[[2-[4-(aminosulfonyl) phenyl] ethyl] amino] carbonyl]benzyl-*N*,*N*-dimethyl-4-fluorobenzylammonium bromide (2)

Following similar procedures as described above for the preparation of **1** by starting with **5** (155 mg, 0.3 mmol) and 4-(2-aminoethyl) benzenesulfonamide (80 mg, 0.4 mmol) in methanol (5 mL), 153 mg (93%) of **2** was obtained as white solid. ^1 ^H NMR (300 MHz, DMSO) δ 8.73 (t, *J* = 5.5 Hz, 1H), 7.94 (d, *J* = 8.2 Hz, 2H), 7.74 (d, *J* = 8.2 Hz, 2H), 7.71–7.60 (m, 4H), 7.53–7.33 (m, 4H), 7.30 (s, 2H), 4.63 (s, 4H), 3.55 (dd, *J* = 12.8, 6.8 Hz, 2H), 2.94 (t, *J* = 7.1 Hz, 2H), 2.88 (s, 6H). MS (ESI) calculated for C_25_H_29_FN_3_O_3_S^+^ 470.2, found (M)^+^ 470.2. Melting point: 215 °C.

#### *N*-4-[[[4-(aminosulfonyl) benzyl] amino] carbonyl] benzyl-*N*,*N*-dimethyl-4-fluorobenzylammonium bromide (3)

Following similar procedures as described above for the preparation of **1** by starting with **5** (155 mg, 0.3 mmol) and 4-(aminomethyl) benzenesulfonamide (80 mg, 0.43 mmol) in methanol (5 mL), 160 mg (99%) of **3** was obtained as white solid. ^1 ^H NMR (300 MHz, DMSO) δ 9.29 (t, *J* = 5.9 Hz, 1H), 8.03 (d, *J* = 8.2 Hz, 2H), 7.77 (d, *J* = 8.3 Hz, 2H), 7.74–7.59 (m, 4H), 7.49 (d, *J* = 8.3 Hz, 2H), 7.37 (t, *J* = 8.8 Hz, 2H), 7.31 (s, 2H), 4.65 (s, 2H), 4.63 (s, 2H), 4.55 (d, *J* = 5.8 Hz, 2H), 2.88 (s, 6H). MS (ESI) calculated for C_24_H_27_FN_3_O_3_S^+^ 456.2, found (M)^+^ 456.0. Melting point: 228 °C.

#### *N*,*N*-dimethyl-*N*-4-[(2,3,5,6-tetrafluorophenoxy) carbonyl] benzyl-4-(4,4,5,5-tetramethyl-1,3,2-dioxaborolan-2-yl)benzylammonium bromide (6)

A mixture of **4** (2.15 g, 6.6 mmol) and 4-(bromomethyl) benzeneboronic acid pinacol ester (1.95 g, 6.6 mmol) in acetonitrile (15 mL) was stirred at room temperature for 20 h. The resulting precipitate was filtered and washed with diethyl ether (10 mL ×3) to yield **6** as white solid (584 mg, 14%). ^1 ^H NMR (300 MHz, DMSO) δ 8.33 (d, *J* = 8.4 Hz, 2H), 8.15–7.98 (m, 1H), 7.90 (d, *J* = 8.3 Hz, 2H), 7.81 (d, *J* = 8.0 Hz, 2H), 7.62 (d, *J* = 8.0 Hz, 2H), 4.77 (s, 2H), 4.68 (s, 2H), 2.95 (s, 6H), 1.31 (s, 12H). MS (ESI) calculated for C_29_H_31_BF_4_NO_4_^+^ 544.2, found (M)^+^ 544.3. Melting point: 179 °C.

#### *N*,*N*-dimethyl-*N*-4-[(2,3,5,6-tetrafluorophenoxy) carbonyl] benzyl-4-(4,4,5,5-tetramethyl-1,3,2-dioxaborolan-2-yl)benzylammoniumtrifluoromethanesulfonate (7)

A solution of silver trifluoromethanesulfonate (232 mg, 0.9 mmol) in acetonitrile (3 mL) was added dropwise to a solution of **6** (562 mg, 0.9 mmol) in a mixture of acetonitrile (6 mL) and methanol (6 mL). The resulting solution was stirred for 4 h. The formed silver bromide precipitate was filtered through celite, and washed with methanol (5 mL ×2). The filtrate was concentrated under reduced pressure to yield **7** as light yellow solid (625 mg, 100%). ^1 ^H NMR (300 MHz, CDCl_3_) δ 8.23 (d, *J* = 8.2 Hz, 2H), 7.87 (d, *J* = 7.9 Hz, 2H), 7.79 (d, *J* = 8.3 Hz, 2H), 7.50 (d, *J* = 7.9 Hz, 2H), 7.16 – 6.94 (m, 1H), 4.95 (s, 2H), 4.75 (s, 2H), 2.99 (s, 6H), 1.34 (s, 12H). MS (ESI) calculated for C_29_H_31_BF_4_NO_4_^+^ 544.2, found (M)^+^ 544.3. MS (ESI) calculated for CF_3_O_3_S^−^ 149.0, found (M)^−^ 149.0. Melting point: 108 °C.

#### *N*-4-[[[4-(aminosulfonyl) benzyl] amino] carbonyl] benzyl-*N*,*N*-dimethyl-4-(4,4,5,5-tetramethyl-1,3,2-dioxaborolan-2-yl)benzylammonium trifluoromethanesulfonate (8)

A mixture of **7** (200 mg, 0.29 mmol) and 4-(2-aminoethyl) benzenesulfonamide (64 mg, 0.32 mmol) in methanol (6 mL) was stirred at room temperature for 20 h. The volatile solvent was removed under reduced pressure. Tetrahydrofuran (6 mL) was added to the residue, and the resulting mixture was sonicated for 5 min. The resulting precipitate was filtered, washed with tetrahydrofuran (1 mL ×2), and dried under reduced pressure to yield **8** as white solid (135 mg, 64%). ^1 ^H NMR (300 MHz, DMSO) δ 8.77–8.66 (m, 1H), 7.93 (d, *J* = 6.9 Hz, 2H), 7.80 (d, *J* = 7.9 Hz, 1H), 7.74 (d, *J* = 8.2 Hz, 2H), 7.71–7.63 (m, 2H), 7.63–7.51 (m, 3H), 7.43 (d, *J* = 8.2 Hz, 2H), 7.30 (s, 2H), 4.75–4.46 (m, 4H), 3.69–3.49 (m, 2H), 3.06–2.90 (m, 2H), 2.89 (d, *J* = 12.6 Hz, 6H), 1.31 (s, 6H). MS (ESI) calculated for C_31_H_41_BN_3_O_5_S ^+^ 578.3, found (M)^+^ 578.4. MS (ESI) calculated for CF_3_O_3_S^−^ 149.0, found (M)^−^ 149.0. Melting point: 165 °C.

### Binding affinity measurement

A stopped-flow method^34^ has been used for assaying the CA catalyzed CO_2_ hydration activity with Phenol red as indicator, working at the absorbance maximum of 557 nm, following the initial rates of the CA-catalyzed CO_2_ hydration reaction for 10–100 s. For each inhibitor at least six traces of the initial 5–10% of the reaction have been used for determining the initial velocity. The uncatalyzed rates were determined in the same manner and subtracted from the total observed rates. Stock solutions of inhibitor (0.01 mM) were prepared in distilled-deionized water with 5% DMSO and dilutions up to 0.1 nM were done thereafter with the assay buffer. The inhibition constant (*K*_i_) was obtained by considering the classical Michaelis–Menten equation, which has been fitted by non-linear least squares by using PRISM 3. All CA isozymes used in the experiments were purified human recombinant proteins obtained as reported earlier by our group^35–45^.

### Radiosynthesis of [^18^F]2

The ^18^F-fluoride in H_2_[^18^O]O was passed through the QMA cartridge, and ^18^F-fluoride was trapped and then eluted out with 0.3 mL aqueous solution of 5 mg tetrabutylammonium triflate into a 4-mL V-shaped reaction vial. Acetonitrile (1 mL) was added and the reaction vial was placed in a heating block and heated at 110 °C under vacuum for 6 min and subsequently under N_2_ flow for another 6 min. A mixture of Cu(OTf)_2_ (100 μL, 0.2 M), pyridine (500 μL, 1 M), and precursor (100 μL, 40 mM) solutions in *N,N*-dimethylformamide was added to the reaction vial. The reaction mixture was incubated at 110 °C for 20 min. The reaction was quenched with water (1 mL) and the resulted mixture was purified by HPLC using the semi-preparative column eluted with 24% CH_3_CN and 76% TEA-PB at a flow rate of 4.5 mL/min. The retention time of ^18^F-labeled **2** was 17.6 min. The collected ^18^F-labeled tracer was diluted with ammonium formate (50 mM, 50 mL), and trapped on a C_18_ light Sep-Pak cartridge. The final product was eluted out with ethanol (0.4 mL), and formulated with saline (4 mL) for plasma stability, biodistribution, and PET/CT imaging studies. Quality control was performed by HPLC on the analytical column eluted with 25% CH_3_CN and 75% PBS at a flow rate of 2.0 mL/min. The retention time of ^18^F-labeled **2** was 6.9 min. The specific activity of ^18^F-labeled **2** was measured using the analytical HPLC system. It was calculated via dividing the injected radioactivity of ^18^F-labeled tracer solution by the amount of the tracer in the injected solution. The amount of the tracer was calculated from the UV absorbance standard curve of non-radioactive **2**.

### LogD_7.4_ measurement

The LogD_7.4_ value of ^18^F-labeled **2** was measured using the shake flask method as previously reported^46^. Briefly, an aliquot (2 μL) of ^18^F-labeled **2** was added to a vial containing 3 mL of n-octanol and 3 mL of phosphate buffer (0.1 M, pH 7.4). The mixture was vortexed for 1 min and then centrifuged at 3000*g* for 10 min. Samples of the n-octanol (1 mL) and buffer (1 mL) layers were taken and counted in a well counter. LogD_7.4_ was calculated using the following equation: LogD_7.4_ = log_10_[(counts in n-octanol phase)/(counts in buffer phase)].

### Stability in mouse plasma

Stability in plasma was performed following published procedures^47^^,^^48^. Aliquots (100 μL) of ^18^F-labeled **2** were incubated with 400 μL of balb/c mouse plasma (Innovative Research, Novi, MI) for upwards of 60 min at 37 °C. At the end of each incubation period, samples were quenched by addition of acetonitrile (0.5 mL), centrifuged to remove proteins, and finally passed through a 0.2 micron filter. The filtered samples were loaded onto the analytical radio-HPLC to check for metabolite formation, and analyses were conducted using Agilent ChemStation software.

### *In vivo* experiments

*In vivo* experiments were conducted in accordance with the guidelines established by the Canadian Council on Animal Care and approved by the Animal Ethics Committee of the University of British Columbia. Male immunodeficient NOD.Cg-*Prkdc^scid^Il2rg^tm1Wjl^*/SzJ (NSG) mice were obtained from a breeding colony at the Animal Resource Centre of the BC Cancer Research Centre.

#### Tumor implantation

Animal model was established following previously published procedures^27^. Under anesthesia with 2.5% isoflurane in 2.0 L/min of oxygen, mice were subcutaneously inoculated with 5 × 10^6^ HT-29 cells (in 100 μL PBS and Matrigel at 1:1 ratio) under the right dorsal flank. Biodistribution studies and PET/CT imaging were performed when tumors reached 7–9 mm in diameter.

#### PET imaging and biodistribution studies

For dynamic imaging study, tumor-bearing mice were sedated with 2% isoflurane inhalation and positioned prone onto the scanner bed. A 10 min baseline CT scan was obtained for localization and attenuation correction before radiotracer injection, using 60 kV X-rays at 500 mA, three sequential bed position with 33% overlap, and 220 degree continuous rotation. PET data were acquired in list mode acquisition, reconstructed using the 3d-OSEM-MAP algorithm with CT-based attenuation correction. The dynamic acquisition of 60 min was started at the time of intravenous injection with 6–8 MBq of the radiotracer. The list mode data were rebinned into time intervals (12 × 10, 6 × 30, 5 × 60, 6 × 300, and 2 × 600 s) to obtain tissue time-activity curves. The mice were kept warm by a heating pad during acquisition. For static imaging study, the mice were briefly sedated for intravenous injection of the radiotracer (6–8 MBq), and allowed to recover and roam freely in their cages for 45 min. At that point, the mice were sedated with 2% isoflurane inhalation, placed on the scanner, and an attenuation correction CT scan was obtained as described above. A single static emission scan was subsequently acquired for 10 min.

Biodistribution studies were performed to confirm the quantitative ROI uptake values observed from PET scans. At 1 h p.i., mice were euthanized. Blood was promptly withdrawn, and the organs/tissues of interest were harvested, rinsed with normal saline, blotted dry, and weighed. The radioactivity of the collected mouse tissues was counted and expressed as the percentage of the injected dose per gram of tissue (%ID/g).

## Results and discussion

As shown in Figure 2, the three cationic sulfonamides **1**–**3** were designed using 4-dimethylaminobenzoic acid as the linker to connect a 4-fluorobenzyl group and a benzenesulfonamide motif for CA-IX targeting. This design also generated the needed cationic quaternary ammonium group that would prevent free diffusion of **1**–**3** into cells and binding to intracellular CA isoforms including CA-I and CA-II. In addition, ^18^F-labeled **1**–**3** could be prepared via copper-mediated aromatic radiofluorination reaction using an arylboronic pinacol ester precursor^49^^,^^50^ as recently demonstrated by us for the preparation 4-[^18^F]fluorobenzyltriphenylphosphonium (^18^F-FBnTP), a myocardial perfusion PET tracer^51^.

**Scheme 1.**
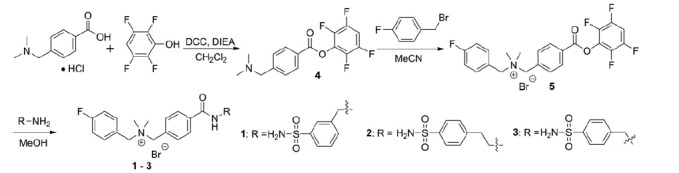
Synthesis of cationic carbonic anhydrase IX inhibitors **1**–**3**.

The preparation of compounds **1**–**3** followed the procedures depicted in Scheme 1. The activated ester **4** was obtained in 99% yield by reacting 4-[(dimethylamino) methyl] benzoic acid with 2,3,5,6-tetrafluorophenol using *N*,*N′*-dicyclohexylcarbodiimide (DCC) as the coupling reagent. Reacting **4** with 4-fluorobenzyl bromide in acetonitrile afforded the quaternary ammonium bromide salt **5** as a precipitate which was isolated in 87% yield by filtration. The desired compounds **1**–**3** were obtained by coupling **5** in methanol with excess 3-(aminomethyl)benzenesulfonamide, 4-(2-aminoethyl)benzenesulfonamide, and 4-(aminomethyl)benzenesulfonamide, respectively. After evaporating methanol, the residue was triturated with tetrahydrofuran to obtain compounds **1**–**3** bromide salt in 93–99% yields.

After synthesis, the binding affinity of compounds **1**–**3** were evaluated for four physiologically relevant CAs: CA-I, -II, -IV, and -IX (Table 1). CA-I and CA-II are cytosolic isozymes that are found primarily in red blood cells, while CA-IV is a glycosyl-phosphatiydyl-inositol anchored membrane isozyme found primarily in the eyes, lungs, and kidneys^6^. Compounds **1**–**3** exhibited different inhibitory profiles. The derivatives have inhibition constants (*K*_i_) in the ranges of 0.52–0.89, 0.07–0.72, 3.63–> 50, and 0.22–0.96 μM for CA-I, -II, -IV, and -IX, respectively. Relative to acetazolamide (*K*_i_(CA-IX) = 0.03 μM), a pan CA inhibitor, the binding affinities of compounds **1**–**3** for CA-IX were substantially lower. With the best affinity towards CA-IX, compound **2** was selected for radiolabeling and *in vivo* experiments.

**Table 1.** Inhibition constants (*K*_i_) of cationic inhibitors 1–3 to carbonic anhydrases I, II, IV, and IX as determined by a stopped-flow CO_2_ hydration assay. Errors in the range of 5–10% of the reported value from three different assays.

	Inhibition constant (*K*_i_, μM)			
Compound	CA-I	CA-II	CA-IV	CA-IX
**1**	0.89	0.72	>50.0	0.44
**2**	0.52	0.07	9.54	0.22
3	0.78	0.48	3.63	0.96
**Acetazolamide**	0.25	0.01	0.08	0.03

**Scheme 2.**
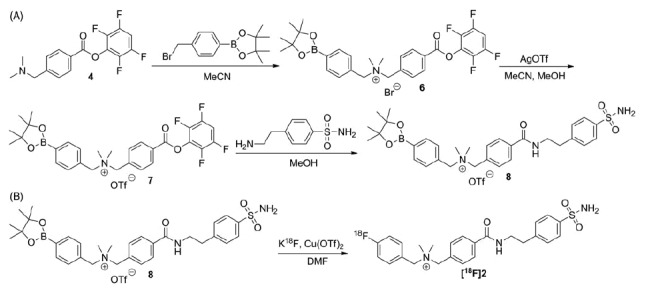
(A) Synthesis of the radiolabeling precursor **8**. (B) Synthesis of ^18^F-labeled **2**.

The preparation of the ^18^F-fluorination precursor **8** is shown in Scheme 2(A). Reacting the activated ester **4** with 4-bromomethylphenylboronic acid pinacol ester afforded the quaternary ammonium bromide salt **6** in 14% yield. The ammonium bromide salt **6** was treated with silver triflate and converted quantitatively to the ammonium triflate salt **7**. The desired precursor **8** was obtained in 64% by coupling **7** with 4-(2-aminoethyl)benzenesulfonamide.

**Figure 3.**
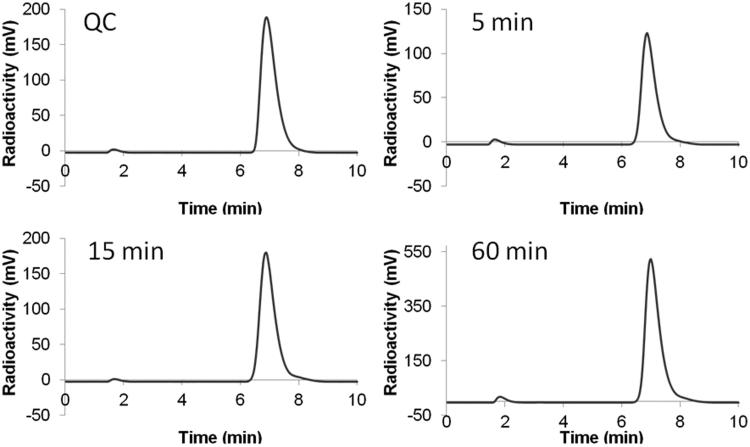
HPLC chromatograms of ^18^F-labeled **2** from (A) QC sample, or plasma sample after being incubated at 37 °C for (B) 5 min, (C) 15 min, or (D) 60 min.

Synthesis of ^18^F-labeled **2** was performed by nucleophilic substitution of the precursor **8** with ^18^F-fluoride via copper-mediated aromatic radiofluorination reaction (Scheme 2(B))^49–51^. Purification and quality control of ^18^F-labeled **2** was performed by HPLC and ^18^F-labeled **2** was isolated in 10 ± 4% (*n* = 3) decay-corrected radiochemical yield with >98% radiochemical purity and 85.1 ± 70.3 GBq/μmol specific activity. An *in vitro* stability study was conducted by incubating ^18^F-labeled **2** at 37 °C in mouse plasma, and monitored by HPLC. As shown in Figure 3, no noticeable degradation of ^18^F-labeled **2** was observed after 60 min incubation, suggesting high stability of ^18^F-labeled **2** in mouse plasma. Lipophilicity of ^18^F-labeled **2** was measured using traditional shake flask method^46^. The obtained LogD_7.4_ (D_7.4_: distribution coefficient between n-octanol and pH 7.4 phosphate buffer) value was —0.79 ± 0.02, indicating that the tracer was hydrophilic.

**Figure 4.**
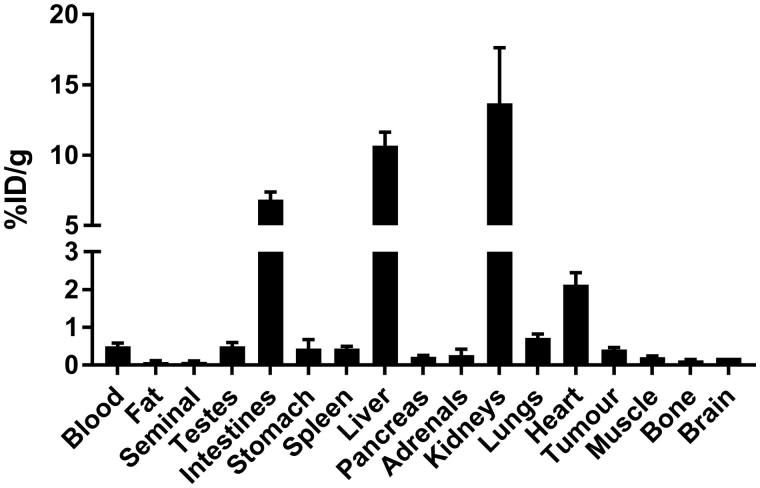
Biodistribution of ^18^F-labeled **2** at 1 h post-injection in HT-29 tumor-bearing mice. Values (%ID/g) are presented as mean ± standard deviation (*n* = 5).

**Figure 5.**
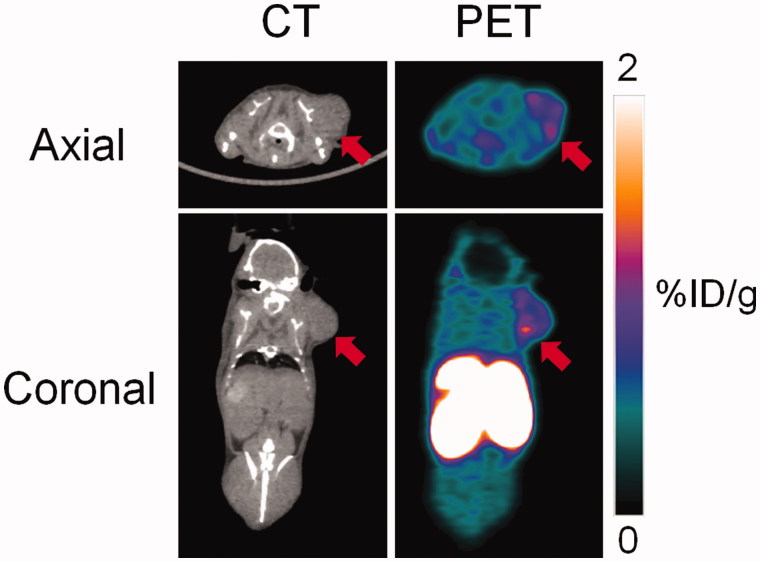
Representative PET and CT images acquired at 1 h post-injection with ^18 ^F-labeled **2** in HT-29 colorectal cancer xenograft-bearing mice. Arrow indicates location of tumor.

**Figure 6.**
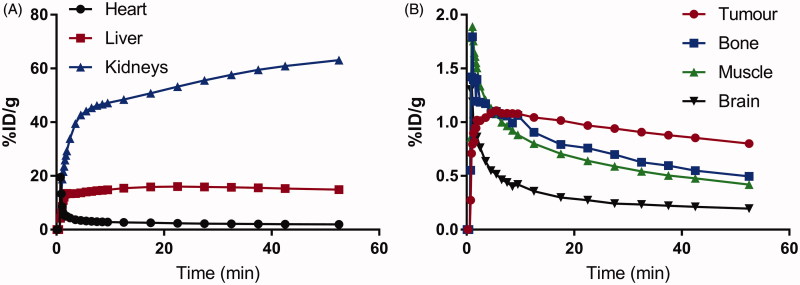
Time activity curves for ^18^F-labeled **2** using ROIs drawn around high activity organs (A) heart, liver, and kidneys, and low activity organs (B) tumor, bone, muscle, and muscle.

*In vivo* imaging experiments were conducted in immunodeficient NSG mice bearing HT-29 human colorectal cancer xenografts. Biodistribution data and representative PET/CT images acquired at 1 h post-injection are shown in Figures 4 and 5, respectively. Tracer uptake was predominantly observed in the excretory organs, liver (10.7 ± 0.96%ID/g) and kidneys (13.7 ± 3.96%ID/g). Moderate uptake was observed in HT-29 tumor xenografts (0.41 ± 0.06%ID/g), which corresponded to tumor-to-muscle ratio of 1.99 ± 0.25. The lowest uptake was observed for the brain (0.02 ± 0.00%ID/g), indicating that the tracer was unable to penetrate the blood–brain barrier. The tracer was stable against *in vivo* defluorination as uptake in bone was observed in negligible amount at 0.13 ± 0.02%ID/g. PET images are consistent with biodistribution data, as the gastrointestinal tract and kidneys showed the highest accumulation of activity. HT-29 xenografts were visualized in PET images with moderate tumor-to-background contrast. Analyzing the time activity curve for ^18^F-labeled **2** (Figure 6), tracer was rapidly cleared through the kidneys and hepatobiliary tract. Despite moderate uptake, the uptake in tumor xenograft was higher compared to non-target tissues like bone, brain, and muscle, enabling its visualization in PET images.

The development of CA-IX targeting agents, particularly those derived from small molecule inhibitors has seen marked improvement in terms of tumor targeting and visualization. Initial efforts were hampered by the lack of target specificity, poor pharmacokinetics, and/or tracer instability^24^^,^^25^. Subsequently, our group and others have leveraged a radiometal-based approach to develop cell-impermeable tracers targeting CA-IX *in vivo*. Most recently, Pomper’s group radiolabeled a dual motif CA-IX inhibitor consisting of a 4,4-bis(4-hydroxyphenyl)valeric acid and a succinyl acetazolamide group with ^111^In (via DOTA chelator) and ^64^Cu (via NOTA chelator) and acheived tumor uptakes values of 14.5–26.0%ID/g at 1 h p.i.^29^^,^^30^. Krall et al.^31^ reported the synthesis and evaluation of a ^99m^Tc-labeled acetazolamide derivative with excellent tumor targeting (22%ID/g at 3 h p.i.) and image contrast. Notably, both research groups used the human renal-cell carcinoma SK-RC-52 model for *in vivo* evaluations. Renal cell carcinomas commonly overexpress CA-IX due to perturbations of the von Hippel-Lindau (*VHL*) gene, which in turn regulates HIF-1α^52–54^. The expression of CA-IX in this model is not necessarily driven by hypoxia.

Beyond the use of radiometals, another major commonality shared by these successful tracers is the high affinity that they exhibit for CA-IX, typically with *K*_i_ values in the low nanomolar range. On the contrary, compound **2** selected for radiolabeling and evaluated in this study had only moderate binding affinity to CA-IX (*K*_i_ = 0.22 μM). A high binding affinity to the target of interest is one of many factors (stability, selectivity, target density, target accessibility, etc.) that determine efficient tumor targeting and accumulation^55^. Future studies leveraging the use of cationic sulfonamides to synthesize diagnostic agents targeting CA-IX require better understanding of the structure activity relationship to improve tracer affinity. The ability to visualize tumor notwithstanding the moderate uptake value suggests that cationic sulfonamides can potentially be used as pharmacophores for CA-IX imaging agents.

## Conclusion

We designed three cationic sulfonamide inhibitors **1**–**3** to potentially target CA-IX for PET applications. Imaging and biodistribution data for ^18^F-labeled **2** showed clear visualization of tumor xenografts despite moderate uptake and tumor-to-background contrast. This is encouraging considering the relatively modest binding affinity of **2** to CA-IX. Therefore, our data demonstrate the use of cationic motif may be useful for designing future CA-IX tracers assuming high affinity binders can be obtained.

## References

[1] Vaupel P, Mayer A. Hypoxia in cancer: significance and impact on clinical outcome. Cancer Metastasis Rev 2007;26:225–39. 17440684 10.1007/s10555-007-9055-1

[2] Walsh JC, Lebedev A, Aten E, et al The clinical importance of assessing tumor hypoxia: relationship of tumor hypoxia to prognosis and therapeutic opportunities. Antioxid Redox Signal 2014;21:1516–54. 24512032 10.1089/ars.2013.5378 PMC4159937

[3] Wilson WR, Hay MP. Targeting hypoxia in cancer therapy. Nature Rev Cancer 2011;11:393–410. 21606941 10.1038/nrc3064

[4] Brown JM. Tumor hypoxia in cancer therapy. Meth Enzymol 2007;435:297–321. 17998060 10.1016/S0076-6879(07)35015-5

[5] Potter C, Harris AL. Hypoxia inducible carbonic anhydrase IX, marker of tumour hypoxia, survival pathway and therapy target. Cell Cycle 2004;3:164–7. 14712082

[6] Alterio V, Di Fiore A, D'Ambrosio K, et al Multiple binding modes of inhibitors to carbonic anhydrases: how to design specific drugs targeting 15 different isoforms? Chem Rev 2012;112:4421–68. 22607219 10.1021/cr200176r

[7] Robertson N, Potter C, Harris AL. Role of carbonic anhydrase IX in human tumor cell growth, survival, and invasion. Cancer Res 2004;64:6160–5. 15342400 10.1158/0008-5472.CAN-03-2224

[8] Hilvo M, Baranauskiene L, Salzano AM, et al Biochemical characterization of CA IX, one of the most active carbonic anhydrase isozymes. J Biol Chem 2008;283:27799–809. 18703501 10.1074/jbc.M800938200

[9] Bartosova M, Parkkila S, Pohlodek K, et al Expression of carbonic anhydrase IX in breast is associated with malignant tissues and is related to overexpression of c-erbB2. J Pathol 2002;197:314–21. 12115877 10.1002/path.1120

[10] Swietach P, Wigfield S, Cobden P, et al Tumor-associated carbonic anhydrase 9 spatially coordinates intracellular pH in three-dimensional multicellular growths. J Biol Chem 2008;283:20473–83. 18482982 10.1074/jbc.M801330200

[11] Swietach P, Wigfield S, Supuran CT, et al Cancer-associated, hypoxia-inducible carbonic anhydrase IX facilitates CO_2_ diffusion. BJU Int 2008;101:22–4. 10.1111/j.1464-410X.2008.07644.x 18430118

[12] Lock FE, McDonald PC, Lou Y, et al Targeting carbonic anhydrase IX depletes breast cancer stem cells within the hypoxic niche. Oncogene 2013;32:5210–19. 23208505 10.1038/onc.2012.550

[13] Lou Y, McDonald PC, Oloumi A, et al Targeting tumor hypoxia: suppression of breast tumor growth and metastasis by novel carbonic anhydrase IX inhibitors. Cancer Res 2011;71:3364–76. 21415165 10.1158/0008-5472.CAN-10-4261

[14] Pacchiano F, Carta F, McDonald PC, et al Ureido-substituted benzenesulfonamides potently inhibit carbonic anhydrase IX and show antimetastatic activity in a model of breast cancer metastasis. J Med Chem 2011;54:1896–902. 21361354 10.1021/jm101541x

[15] Touisni N, Maresca A, McDonald PC, et al Glycosyl coumarin carbonic anhydrase IX and XII inhibitors strongly attenuate the growth of primary breast tumors. J Med Chem 2011;54:8271–7. 22077347 10.1021/jm200983e

[16] Krall N, Pretto F, Decurtins W, et al A small-molecule drug conjugate for the treatment of carbonic anhydrase IX expressing tumors. Angew Chem Int Ed Engl 2014;53:4231–5. 24623670 10.1002/anie.201310709

[17] Cazzamalli S, Dal Corso A, Neri D. Linker stability influences the anti-tumor activity of acetazolamide-drug conjugates for the therapy of renal cell carcinoma. J Control Rel 2017;246:39–45. 10.1016/j.jconrel.2016.11.023 PMC5266555 27890855

[18] Wichert M, Krall N. Targeting carbonic anhydrase IX with small organic ligands. Curr Opin Chem Biol 2015;26:48–54. 25721398 10.1016/j.cbpa.2015.02.005

[19] Apte SD, Chin FT, Graves EE. Synthesis of a new PET radiotracer targeting carbonic anhydrase IX. J Labelled Comp Radiopharm 2009;52:S408.

[20] Lu G, Hillier SM, Maresca KP, et al Synthesis and SAR of novel Re/99mTc-labeled benzenesulfonamide carbonic anhydrase IX inhibitors for molecular imaging of tumor hypoxia. J Med Chem 2013;56:510–20. 23234246 10.1021/jm3015348

[21] Akurathi V, Dubois L, Celen S, et al Development and biological evaluation of (9)(9)mTc-sulfonamide derivatives for in vivo visualization of CA IX as surrogate tumor hypoxia markers. Eur J Med Chem 2014;71:374–84. 24378650 10.1016/j.ejmech.2013.10.027

[22] Akurathi V, Dubois L, Lieuwes NG, et al Synthesis and biological evaluation of a 99mTc-labelled sulfonamide conjugate for in vivo visualization of carbonic anhydrase IX expression in tumor hypoxia. Nuclear Med Biol 2010;37:557–64. 10.1016/j.nucmedbio.2010.02.006 20610160

[23] Turkbey B, Lindenberg ML, Adler S, et al PET/CT imaging of renal cell carcinoma with (18)F-VM4-037: a phase II pilot study. Abdom Radiol (NY) 2016;41:109–18. 26830617 10.1007/s00261-015-0599-1 PMC7788639

[24] Pan J, Lau J, Mesak F, et al Synthesis and evaluation of 18F-labeled carbonic anhydrase IX inhibitors for imaging with positron emission tomography. J Enzyme Inhib Med Chem 2014;29:249–55. 23463940 10.3109/14756366.2013.773994

[25] Lau J, Pan J, Zhang Z, et al Synthesis and evaluation of (18)F-labeled tertiary benzenesulfonamides for imaging carbonic anhydrase IX expression in tumours with positron emission tomography. Bioorg Med Chem Lett 2014;24:3064–8. 24878197 10.1016/j.bmcl.2014.05.021

[26] Lau J, Liu Z, Lin KS, et al Trimeric radiofluorinated sulfonamide derivatives to achieve in vivo selectivity for carbonic anhydrase IX-targeted pet imaging. J Nucl Med 2015;56:1434–40. 26205302 10.2967/jnumed.114.153288

[27] Lau J, Zhang Z, Jenni S, et al PET imaging of carbonic anhydrase IX expression of HT-29 tumor xenograft mice with (68)Ga-labeled benzenesulfonamides. Molec Pharmaceut 2016;13:1137–46. 10.1021/acs.molpharmaceut.5b00934 26866675

[28] Sneddon D, Niemans R, Bauwens M, et al Synthesis and in vivo biological evaluation of (68)Ga-labeled carbonic anhydrase IX targeting small molecules for positron emission tomography. J Med Chem 2016;59:6431–43. 27322137 10.1021/acs.jmedchem.6b00623

[29] Yang X, Minn I, Rowe SP, et al Imaging of carbonic anhydrase IX with an 111In-labeled dual-motif inhibitor. Oncotarget 2015;6:33733–42. 26418876 10.18632/oncotarget.5254 PMC4741798

[30] Minn I, Koo SM, Lee HS, et al [64Cu]XYIMSR-06: a dual-motif CAIX ligand for PET imaging of clear cell renal cell carcinoma. Oncotarget 2016;7:56471–9. 27437764 10.18632/oncotarget.10602 PMC5302928

[31] Krall N, Pretto F, Mattarella M, et al A technetium 99m-labeled ligand of carbonic anhydrase IX selectively targets renal cell carcinoma in vivo. J Nucl Med 2016;6:943–9. 10.2967/jnumed.115.170514 26912427

[32] Casey JR, Morgan PE, Vullo D, et al Carbonic anhydrase inhibitors. Design of selective, membrane-impermeant inhibitors targeting the human tumor-associated isozyme IX. J Med Chem 2004;47:2337–47. 15084132 10.1021/jm031079w

[33] Basavaraj S, Betageri GV. Can formulation and drug delivery reduce attrition during drug discovery and development-review of feasibility, benefits and challenges. Acta Pharmaceut Sinica B 2014;4:3–17. 10.1016/j.apsb.2013.12.003 PMC4590717 26579359

[34] Khalifah RG. The carbon dioxide hydration activity of carbonic anhydrase. I. Stop-flow kinetic studies on the native human isoenzymes B and C. J Biol Chem 1971;246:2561–73. 4994926

[35] Yamali C, Gul HI, Sakagami H, Supuran CT. Synthesis and bioactivities of halogen bearing phenolic chalcones and their corresponding bis Mannich bases. J Enzyme Inhib Med Chem 2016;31:125–31. 27594305 10.1080/14756366.2016.1221825

[36] Mollica A, Locatelli M, Macedonio G, et al Microwave-assisted extraction, HPLC analysis, and inhibitory effects on carbonic anhydrase I, II, VA, and VII isoforms of 14 blueberry Italian cultivars. J Enzyme Inhib Med Chem 2016;31 1–16. 10.1080/14756366.2016.1214951 27541737

[37] Margheri F, Ceruso M, Carta F, et al Overexpression of the transmembrane carbonic anhydrase isoforms IX and XII in the inflamed synovium. J Enzyme Inhib Med Chem 2016;31:60–3. 27539792 10.1080/14756366.2016.1217857

[38] Mishra CB, Kumari S, Angeli A, et al Design, synthesis and biological evaluation of N-(5-methyl-isoxazol-3-yl/1,3,4-thiadiazol-2-yl)-4-(3-substitutedphenylureido) benzenesulfonamides as human carbonic anhydrase isoenzymes I, II, VII and XII inhibitors. J Enzyme Inhib Med Chem 2016;31:174–9. 27314170 10.1080/14756366.2016.1197221

[39] Diaz JR, Fernandez Baldo M, Echeverria G, et al A substituted sulfonamide and its Co (II), Cu (II), and Zn (II) complexes as potential antifungal agents. J Enzyme Inhib Med Chem 2016;31:51–62. 27232977 10.1080/14756366.2016.1187143

[40] Supuran CT, Kalinin S, Tanc M, et al Isoform-selective inhibitory profile of 2-imidazoline-substituted benzene sulfonamides against a panel of human carbonic anhydrases. J Enzyme Inhib Med Chem 2016;31:197–202. 27160030 10.1080/14756366.2016.1178248

[41] Federici C, Lugini L, Marino ML, et al Lansoprazole and carbonic anhydrase IX inhibitors sinergize against human melanoma cells. J Enzyme Inhib Med Chem 2016;31:119–25. 10.1080/14756366.2016.1177525 27142956

[42] Chohan ZH, Scozzafava A, Supuran CT. Unsymmetrical 1,1'-disubstituted ferrocenes: synthesis of Co(ii), Cu(ii), Ni(ii) and Zn(ii) chelates of ferrocenyl -1-thiadiazolo-1'-tetrazole, -1-thiadiazolo-1'-triazole and -1-tetrazolo-1'-triazole with antimicrobial properties. J Enzyme Inhib Med Chem 2002;17:261–6. 12530479 10.1080/1475636021000006261

[43] Del Prete S, Vullo D, Fisher GM, et al Discovery of a new family of carbonic anhydrases in the malaria pathogen Plasmodium falciparum – the eta-carbonic anhydrases. Bioorg Med Chem Lett 2014;24:4389–96. 25168745 10.1016/j.bmcl.2014.08.015

[44] Supuran CT, Scozzafava A, Mastrolorenzo A. Bacterial proteases: current therapeutic use and future prospects for the development of new antibiotics. Expert Opin Ther Pat 2001;11:221–59.

[45] Supuran CT, Barboiu M, Luca C, et al Carbonic anhydrase activators. 14. Syntheses of mono and bis pyridinium salt derivatives of 2-amino-5-(2-aminoethyl)- and 2-amino-5-(3-aminopropyl)-1,3,4-thiadiazole and their interaction with isozyme II. Eur J Med Chem 1996;31:597–606.

[46] Lin KS, Pan J, Amouroux G, et al In vivo radioimaging of bradykinin receptor b1, a widely overexpressed molecule in human cancer. Cancer Res 2015;75:387–93. 25488751 10.1158/0008-5472.CAN-14-1603

[47] Amouroux G, Pan J, Jenni S, et al Imaging bradykinin B1 receptor with 68Ga-labeled [des-Arg10]Kallidin derivatives: effect of the linker on biodistribution and tumor uptake. Molec Pharmaceut 2015;12:2879–88. 10.1021/acs.molpharmaceut.5b00070 26101793

[48] Liu Z, Amouroux G, Zhang Z, et al (18)F-trifluoroborate derivatives of [des-arg(10)]kallidin for imaging bradykinin b1 receptor expression with positron emission tomography. Mol Pharmaceut 2015;12:974–82. 10.1021/acs.molpharmaceut.5b00003 25629412

[49] Tredwell M, Preshlock SM, Taylor NJ, et al A general copper-mediated nucleophilic 18F fluorination of arenes. Angew Chem Int Ed Engl 2014;53:7751–5. 24916101 10.1002/anie.201404436

[50] Mossine AV, Brooks AF, Makaravage KJ, et al Synthesis of [18F]Arenes via the copper-mediated [18F]fluorination of boronic acids. Organic Lett 2015;17:5780–3. 10.1021/acs.orglett.5b02875 PMC4672358 26568457

[51] Zhang Z, Zhang C, Lau J, et al One-step synthesis of 4-[(18) F]fluorobenzyltriphenylphosphonium cation for imaging with positron emission tomography. J Labelled Comp Radiopharm 2016;59:467–71. 27578168 10.1002/jlcr.3436

[52] Stillebroer AB, Mulders PF, Boerman OC, et al Carbonic anhydrase IX in renal cell carcinoma: implications for prognosis, diagnosis, and therapy. Eur Urol 2010;58:75–83. 20359812 10.1016/j.eururo.2010.03.015

[53] Chrastina A, Zavada J, Parkkila S, et al Biodistribution and pharmacokinetics of 125I-labeled monoclonal antibody M75 specific for carbonic anhydrase IX, an intrinsic marker of hypoxia, in nude mice xenografted with human colorectal carcinoma. Int J Cancer 2003;105:873–81. 12767076 10.1002/ijc.11142

[54] Ivanov SV, Kuzmin I, Wei MH, et al Down-regulation of transmembrane carbonic anhydrases in renal cell carcinoma cell lines by wild-type von Hippel-Lindau transgenes. Proc Natl Acad Sci USA 1998;95:12596–601. 9770531 10.1073/pnas.95.21.12596 PMC22876

[55] Srinivasarao M, Galliford CV, Low PS. Principles in the design of ligand-targeted cancer therapeutics and imaging agents. Nature Rev Drug Discov 2015;14:203–19. 25698644 10.1038/nrd4519

